# Total Endovascular Aortic Arch Repair Using In Situ Needle Triple Fenestration and Selective Cerebral Perfusion: Single-Center Results

**DOI:** 10.3390/jcm14186377

**Published:** 2025-09-10

**Authors:** Evren Ozcinar, Fatma Akca, Mehmet Cahit Saricaoglu, Ali Ihsan Hasde, Nur Dikmen, Onur Buyukcakir, Aysegul Guven, Oguzhan Durmaz, Salih Anil Boga, Ali Fuat Karacuha, Melisa Kandemir, Levent Yazicioglu, Sadik Eryilmaz

**Affiliations:** 1 Department of Cardiovascular Surgery, Ankara University, 06100 Ankara, Turkey; evrenozcinar@gmail.com (E.O.); ahasde@gmail.com (A.I.H.); nurdikmen@yahoo.com (N.D.); buyukcakironur@gmail.com (O.B.); melisakandemir1999@gmail.com (M.K.); leventyazicioglu@hotmail.com (L.Y.); sadikeryilmaz@gmail.com (S.E.); 2 Department of Cardiovascular Surgery, Kirikkale High Specialization Hospital, 71300 Kirikkale, Turkey; akcaafatma@gmail.com (F.A.); anilboga@hotmail.com (S.A.B.); 3 Department of Interdisciplinary Food, Metabolism and Clinical Nutrition, Institute of Health Sciences, Ankara University, 06230 Ankara, Turkey; 4 Department of Anesthesiology and Reanimation, Ankara University Faculty of Medicine, 06230 Ankara, Turkey; guven_aysegul@hotmail.com; 5 Department of Cardiovascular Surgery, Perfusion Unit, Ankara University Faculty of Medicine, 06230 Ankara, Turkey; durmazogz@gmail.com; 6 Department of Cardiovascular Surgery, Trabzon Kanuni Training and Research Hospital, 61040 Trabzon, Turkey; alifuatkaracuha@hotmail.com

**Keywords:** thoracic endovascular aortic repair, in situ needle fenestration, aortic arch, total endovascular aortic arch replacement, cardiopulmonary bypass, cerebral perfusion

## Abstract

**Background:** Advances in stent grafts and endovascular techniques have expanded the indications for thoracic endovascular aortic repair (TEVAR) to include arch lesions. In situ needle fenestration (ISNF) has emerged as a promising technique for revascularizing supra-aortic branches. The aim of this study is to evaluate the safety and efficacy of triple in situ needle fenestration during TEVAR for aortic arch pathologies in a single-center experience. **Materials and Methods:** A retrospective analysis was conducted on fifteen patients who underwent in situ triple fenestration TEVAR between June 2023 and March 2024. The median age of the patients was 51,33 years (±19.69) and twelve of the patients were male. All procedures were performed under general anesthesia in a hybrid operating room. Ethical approval was obtained from the institutional review board, and informed consent was received from all participants. **Results:** Primary technical success was achieved in all cases (15/15, 100%). The mean operation time was 197.33 min (range: 126–302). Two patients experienced a minor hematoma at the access site. Mortality was observed in one patient (6.66%) during the 30-day follow-up period. The total hospital stay averaged 7 ± 3.36 days. One patient had a transient ischemic attack, but there were no incidents of stroke or spinal cord ischemia. No procedure-related endoleak was observed during the intervention; however, eight patients required reintervention in the descending aorta. **Conclusions:** ISNF may be an effective and feasible method for revascularizing arch vessels, with low rates of early mortality and stroke when performed by experienced practitioners. However, larger multicenter studies with longer follow-up are needed to confirm the durability and long-term outcomes of this technique.

## 1. Introduction

Aortic arch pathologies continue to challenge surgeons due to the complexity of surgical management. Although conventional surgical repair, which involves cardiopulmonary bypass and deep circulatory arrest, is considered the gold standard, it is associated with high mortality and morbidity. In patients with significant comorbidities, particularly in redo cases with a history of ascending aorta surgery, hybrid techniques have become preferable [1,2]. To reconstruct the supra-aortic arteries, branched TEVAR (Thoracic Endovascular Aortic Repair), chimney techniques, and fenestration techniques have been developed. These techniques also have disadvantages, such as technical difficulty, high cost, and the need for advanced expertise [1]. Therefore, there is a continuous search for new and improved approaches.

In our study, the indications for treatment included aortic aneurysms (5 patients) and aortic dissections (10 patients). Among the dissection patients, all had a history of prior ascending aorta surgery, such as Bentall procedures or tubular graft implantation, and required intervention due to residual dissection. These indications align with the literature, which highlights the complexity of managing aortic arch pathologies in patients with prior surgical interventions [3,4].

While the chimney technique is commonly used for visceral branches, fenestration techniques are widely applied in TEVAR cases where the landing zone is insufficient and the left subclavian artery needs to be covered. Revascularization of the left subclavian artery has been shown to reduce cerebrovascular accidents, spinal cord ischemia, and left upper extremity ischemia [3]. The anatomical challenges and neurological risks associated with surgical revascularization for Zone 2 TEVAR and aortic arch pathologies have encouraged the exploration of alternative endovascular solutions. In situ fenestration (ISF) of thoracic endografts is the most common endovascular alternative to open repair techniques such as carotico-subclavian bypass. The development of self-centering, adjustable, needle-based puncture devices has improved the safety and success rates of ISF, addressing previous technical and device-related issues reported with in situ laser fenestration [1,3,4].

Recent studies have demonstrated that in situ fenestration techniques, particularly those using needle-based systems, offer a safer and more effective alternative for aortic arch revascularization. For example, Piffaretti et al. [3] reported high technical success rates and low complication rates with adjustable needle puncture systems, emphasizing the importance of patient selection and procedural planning. These findings are consistent with our clinical experience, where careful preoperative planning and the use of advanced devices have contributed to favorable outcomes.

The aim of this study is to present the outcomes of triple in situ fenestration for aortic arch revascularization using a specially designed puncture needle in selected patients.

## 2. Methods

### 2.1. Study Design

This is a single-center retrospective analysis of fifteen patients with aortic arch pathologies who underwent in situ triple fenestration TEVAR between June 2023 and March 2024 at the Department of Cardiovascular Surgery, Ankara University.

The study was approved by the institutional review board of Ankara University (approval date: 21 March 2025, protocol number: 2025000227-2, registration number: 2025/227) and was conducted in accordance with the Declaration of Helsinki. Informed consent was obtained from all individual participants included in the study.

All patients underwent follow-up computed tomography angiography (CTA) and clinical evaluations at 1 week, 30 days, 3 months, and 6 months after discharge. Clinical data were obtained from the hospital’s electronic medical records.

Exclusion criteria [1,3]:

Supra-aortic anatomic variants or carotid/vertebral artery stenosis;

History of cerebrovascular events within the past 3 months;

Allergy to iodinated contrast agents;

Lack of available peripheral arterial access;

Life expectancy less than 1 year, severe organ failure, or malignant tumors.

### 2.2. Preoperative Planning and Endovascular Procedure

**Figure 1:**
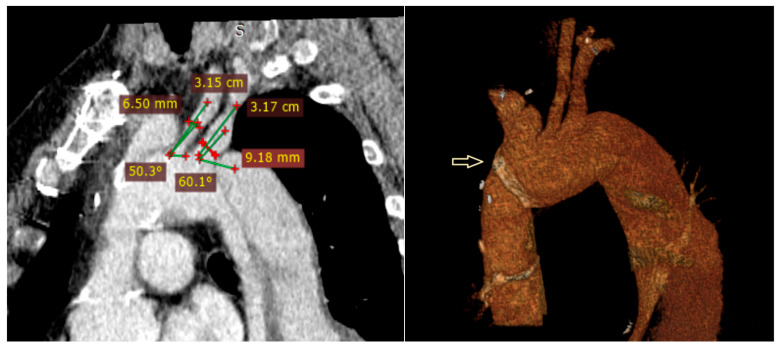
Preoperative CTA coronal section measurements of the diameter of the left carotid and subclavian arteries and the angle formed with the arch, three-dimensional images of the aortic arch, and the suture line from the previous operation (arrow).

Preoperative planning included requesting CT images with the thinnest possible slices (preferably 0.625 mm) to ensure high-quality imaging. The inner and outer diameters of the peripheral vessels used for access, as well as the distances from the access site to the target vessels, were measured. The aortic neck length, diameter, and the angles with the supra-aortic branches were evaluated using multiplanar projections [3]. In Figure 1, the preoperative tomographic measurements and 3D angiography of a patient are presented as an example.

In patients with a history of prior aortic valve repair, special attention was given to the placement of the TEVAR wire to avoid damaging the prosthetic valve. The wire was manipulated under fluoroscopic and transesophageal echocardiographic (TOE) guidance to ensure precise placement without exerting pressure on the valve prosthesis. This approach is supported by the literature, which emphasizes the importance of careful wire management in patients with prosthetic valves [5].

#### 2.2.1. Preparation and Perfusion Strategy

**Figure 2:**
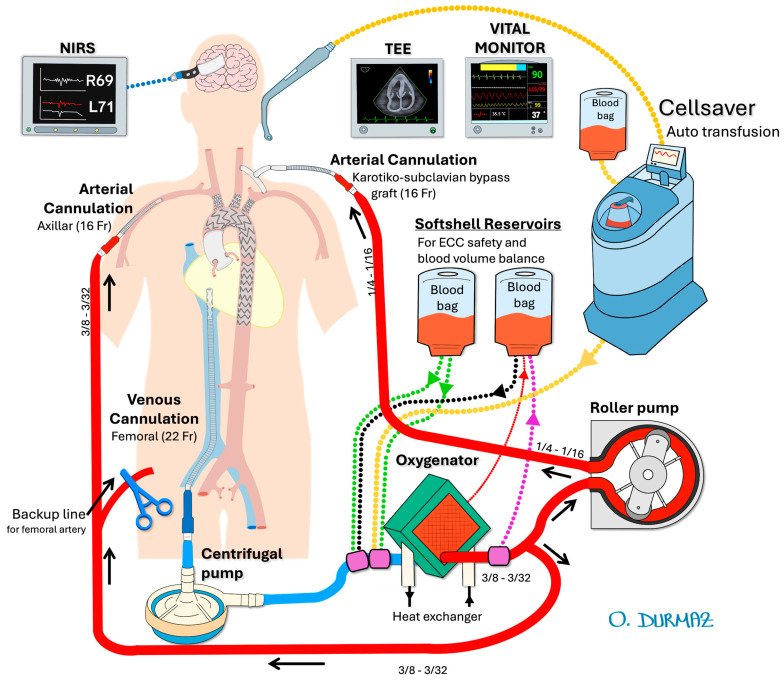
Details of selective cerebral bypass circuit configuration [5]. The circuit ensures continuous perfusion to the brain by directing blood flow through the left carotid–subclavian bypass and the right axillary artery. Cannulas are strategically placed to maintain adequate cerebral oxygenation and minimize ischemic risks throughout the operation. The flow rate and circuit pressure are carefully monitored to optimize perfusion efficiency.

**Figure 3:**
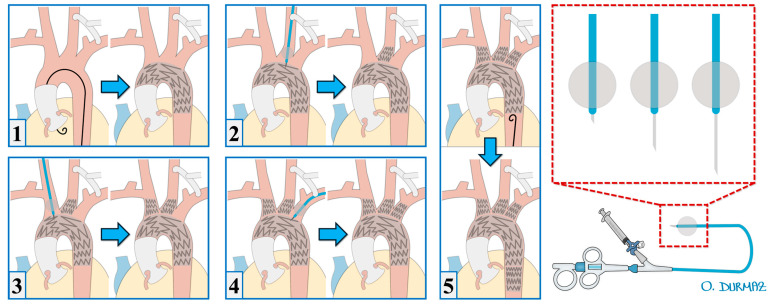
Operative details of the in situ fenestration [5]. The tool kit used for in situ needle fenestration, including the balloon at its tip and the needle system, has been illustrated visually (last image). The wire is placed into the ascending aorta, and the stent graft is deployed in the aortic arch (**1**). Under selective cerebral perfusion, the left common carotid artery is first fenestrated, and a stent is deployed (**2**). Subsequently, the right brachiocephalic artery is fenestrated, and a stent is placed (**3**). The fenestration of the left subclavian artery is performed, completing the fenestration of the aortic arch (**4**). An additional stent graft is then deployed in the descending aorta (**5**).

In our previously published articles, we have described the surgical technique in detail [5,6]. In the mentioned case report, the configuration of cerebral perfusion strategies is illustrated in Figure 2, while the details of the intraoperative endovascular procedure are depicted in Figure 3.

All patients had a cerebrospinal fluid catheter placed by a neurosurgeon under general anesthesia, and cerebrospinal fluid pressure was maintained below 15 mmHg throughout the operation. Transesophageal echocardiography (TOE) was used to ensure accurate deployment of the stent graft. Regional cerebral oxygen saturation was monitored using near-infrared spectroscopy (INVOS 5100C, Somanetics, Troy, MI, USA).

For vascular access, bilateral femoral and carotid arteries, the right axillary artery, and the left brachial artery were exposed via cutdown. To ensure antegrade perfusion, a left carotid-subclavian bypass was performed using an 8 mm Dacron graft. For extra-anatomic perfusion, bilateral selective cerebral perfusion was achieved using a roller pump and a dedicated Dacron graft. The left carotid-subclavian bypass provided antegrade perfusion to the left subclavian artery, while the right axillary artery was cannulated to support additional perfusion. This approach was selected to reduce the risk of cerebrovascular complications and to maintain sufficient perfusion to the brain and upper extremities, as supported by recent studies [6,7].

A 16-French arterial cannula was inserted into the interposed carotico-subclavian bypass graft, and another into the right axillary artery. A 20-French venous cannula was placed into the femoral vein, extending to the right atrium. Heparinization was performed to achieve an activated clotting time > 350 s. Selective cerebral perfusion was maintained at a flow rate of 10 mL/kg/min, with a circuit pressure of 100–150 mmHg and a blood temperature of 34 °C.

#### 2.2.2. Endovascular Aortic Arch In Situ Fenestration Technique

The Ankura (Lifetech Scientific, Shenzhen, China) thoracic stent graft was used in all cases because its flexibility and compatibility with the puncture system have been demonstrated in clinical studies [3,6] which is also supported by our own clinical experience. After initiating selective cerebral perfusion and, if necessary, rapid pacing, the stent graft was deployed. The left common carotid artery and right carotid artery were punctured using a self-centering balloon catheter and an adjustable 20-gauge needle (FuThrough; Lifetech Scientific, Shenzhen, China) via the Seldinger technique.

For left subclavian artery fenestration, a steerable sheath (Fustar; Lifetech Scientific, Shenzhen, China) was placed in the left brachial artery, and fenestration was performed by direct contact with the outer curvature of the stent. After fenestration, a 0.018 or 0.035 guidewire was advanced into the ascending aorta. Balloon dilation was performed, and chrome-cobalt-ePTFE stent grafts (BeGraft; Bentley, Hechingen, Germany), oversized by 10%, were implanted into each arch branch.

**Figure 4:**
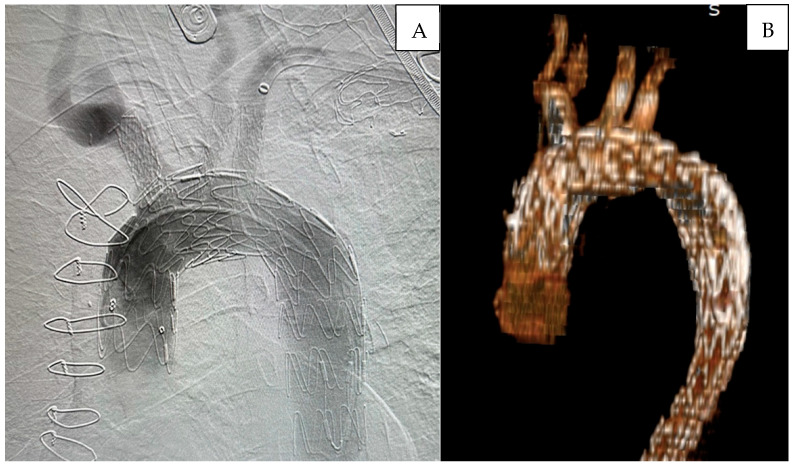
Postoperative digital subtraction angiography (**A**), The 3-D image of the angiography (**B**).

After confirming satisfactory bilateral carotid and vertebral blood flow, selective cerebral extracorporeal circulation was gradually discontinued while ensuring adequate near-infrared spectroscopy levels. Subsequently, control angiography was performed to assess the patency of the arch arteries and to check for any endoleaks. The post-procedural follow-up digital subtraction angiography (DSA) and the 3D reconstruction of angiographic imaging for a patient are demonstrated in Figure 4.

### 2.3. Statistical Analysis

Continuous data are expressed as mean ± standard deviation or median (minimum–maximum), depending on the distribution. Statistical analyses were performed using SPSS software (version 29.0.2, IBM Corp., Armonk, NY, USA). A *p*-value < 0.05 was considered statistically significant.

## 3. Results

Technical success was achieved in all fifteen patients (100%), and all procedures were completed as planned. Twelve of all patients were male, with a mean age of 51.33 ± 19.69 years. All cases were elective. No emergency cases were included due to the need for detailed preoperative planning. Five patients had an aortic aneurysm, while the others had aortic dissection. Among the dissection patients, all had a history of prior ascending aorta surgery, such as tubular graft implantation in the ascending aorta or the Bentall procedure, and underwent endovascular intervention in the aortic arch due to residual dissection (Table 1).

Some outcomes associated with the procedure are presented in Table 2. During the early postoperative intensive care monitoring period, one patient experienced cardiac arrest and despite resuscitation efforts, the patient unfortunately passed away. The exact cause of death could not be definitively determined.

The total operation time, which includes the duration from when the patient is placed on the operating table to their transfer to the intensive care unit, was on average 197.33 min (ranging from 126 to 302 min). The average hospital stay for the patients was 7 ± 3.36 days. In cases that were all elective, preoperative hemoglobin levels were targeted to be high, with an average of 13.58 ± 2.11 g/deciliter. There was no significant bleeding during the procedure, and postoperative levels were found to be 10.92 ± 2.08 g/dL.

**Figure 5:**
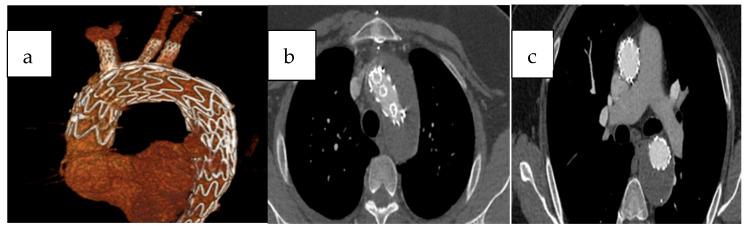
A contrast-enhanced computed tomography (CT) image obtained 6 months after the procedure with volume-rendered 3D reconstruction (**a**) The original axial image clearly demonstrates the contrast enhancement of the stents in the triple arch branches (**b**) and the thrombosed false lumen in the descending aorta (**c**).

Postoperative digital subtraction angiography (DSA) revealed no endoleak at the fenestration site related to the procedure, and no reintervention was required for the aortic arch during follow-up. Figure 5 demonstrates a 6-month follow-up computed tomography scan highlighting the procedural success. Postoperative digital subtraction angiography (DSA) revealed no endoleak at the fenestration site related to the procedure, and no reintervention was required for the aortic arch during follow-up. Appropriate contrast enhancement is observed within the stents of the fenestrated arch branches, and the false lumen in the descending aorta is completely thrombosed.

During postoperative follow-up CT scans, no cases of stent graft migration or Type I endoleak were observed in our original cohort. However, in 8 patients with a history of prior ascending aortic interventions, follow-up CT angiographies revealed insufficient thrombosis and progressive expansion of the false lumen in the descending aorta, necessitating reintervention. To address this issue, the true lumen was reinforced using the STABILISE technique, which involves stent-assisted balloon-induced intimal disruption and relamination. This approach aims to promote complete thrombosis of the false lumen, restore aortic wall integrity, and prevent further complications by remodeling the aortic architecture.

One patient developed a small hematoma at the brachial incision site, which was managed conservatively with compression and resolved without further intervention. The second patient experienced a minor hematoma at the femoral access site, which also resolved spontaneously without requiring surgical intervention. Both cases were closely monitored during the postoperative period, and no long-term sequelae were observed. No other access site complications were observed at the carotid, or axillary access sites (Table 2).

One patient experienced a transient ischemic attack (TIA) and the TIA resolved completely without permanent neurological deficit. No cases of permanent stroke were observed. No cases of spinal cord ischemia, major bleeding complications, or device-related failures were observed during the perioperative period. No patients required conversion to open surgical repair (Table 2).

## 4. Discussion

Surgical interventions for aortic arch pathologies, such as aneurysms and dissections, are complex due to the involvement of critical vessels supplying the brain and upper extremities. This complexity contributes to the high mortality and morbidity rates associated with aortic arch interventions. Isolated aortic arch pathologies are rare and often occur as part of a more extensive disease involving the ascending or descending aorta. Reintervention may be required after open surgery for Type A dissections or following TEVAR due to retrograde dissections [7].

Recent literature indicates that, in 10% to 50% of TEVAR procedures, the intervention may need to be extended proximally, often necessitating the intentional occlusion of the subclavian artery [2,6,8]. Revascularization of the left subclavian artery during TEVAR remains controversial. In the first United States regulatory trial [9], it advocated prophylactic revascularization for all patients if the LSA is to be occluded during TEVAR, but this has not been fully adopted in clinical practice. While some surgeons perform routine prophylactic revascularization, others choose to perform revascularization only in selected patients. Additionally, some apply revascularization post-procedurally in clinical practice if symptoms of left arm ischemia or subclavian steal syndrome occur following TEVAR [10,11,12].

In our study, we performed left carotid-subclavian bypass in all patients to ensure antegrade perfusion to the left subclavian artery. This approach was combined with in situ fenestration to optimize cerebral and upper extremity perfusion. Although this combination may increase procedural complexity, it was chosen to minimize the risk of cerebrovascular complications, as supported by recent studies [3,6].

Alternative endovascular methods, such as chimney, modified endografts, branched or fenestrated endografts, and ISF, have recently contributed to the literature as alternatives to traditional methods like transposition or left carotid-subclavian bypass for LSA revascularization [3,6,13]. Our extra-anatomic perfusion strategy, which involved bilateral selective cerebral perfusion using a roller pump and a dedicated Dacron graft, was designed to minimize ischemic complications. This method aligns with the findings of Sonesson et al. [14], who reported favorable outcomes with bilateral carotid cannulation and centrifugal pump support for cerebral perfusion.

After his in vivo and in vitro studies, Richard G McWilliams published the first clinical case report on the in situ stent-graft technique for LSA revascularization during TEVAR in 2004, and subsequent studies in this area have continued to increase [15,16]. Various techniques such as needle, laser, and radiofrequency fenestration have been described, and short-term successful outcomes with low fenestration-related morbidity rates have been published. This topic continues to remain important in the literature [6,17]. The question of which technique is superior to another remains a gray area. In our clinical practice, we use needle fenestration. Another important factor affecting the success of fenestration is patient selection and stent choice. Patient selection should be made carefully, considering significant studies [3,18] in the literature that define anatomical suitability. With this in mind, and as we detailed in the case report [5], we published as the first in Turkey, we prefer to use the AnkuraTM device (Lifetech Scientific, Shenzhen, China). The stent’s thin double-layer structure made with expanded polytetrafluoroethylene (ePTFE) and the significant flexibility provided by its supporting nitinol layer are its most important features, allowing it to adapt optimally to the curvature of the arch [5,6].

Surgical management of aortic arch pathologies, particularly those requiring circulatory arrest and cerebral protection, remains highly complex and technically demanding. Although total arch replacement performed under deep hypothermia (<18 °C) was introduced in the 1970s to minimize cellular injury, this approach was associated with a significant risk of neurological complications. More recently, optimal cerebral protection strategies—such as the combination of antegrade and retrograde perfusion—have been shown to further reduce perioperative risk [19]. Despite these advances, open arch surgery continues to pose substantial challenges and requires considerable surgical expertise, prompting increased interest in endovascular alternatives.

The literature on endovascular repair of the aortic arch is rapidly expanding. For example, Sonesson et al. [14] reported favorable early outcomes with TEVAR in patients with residual dissection following ascending aorta replacement, utilizing bilateral carotid cannulation and centrifugal pump support for cerebral perfusion. In our series, we adopted a similar approach by providing simultaneous perfusion to both the left carotid artery and the left subclavian artery using a roller pump and a dedicated Dacron graft, aiming to minimize cerebrovascular complications through bilateral selective perfusion.

Our initial experience with total endovascular arch repair in a small cohort has been encouraging, demonstrating high technical success, low complication rates, and short hospital stays. However, the lack of long-term follow-up data remains a limitation, and further studies with larger patient populations are warranted to validate these findings.

### Study Limitations

The fact that the study is a single-center, retrospective study and the potential generalization of the results are important limiting points. Reflecting early outcomes in a small patient population and the need for long-term follow-ups are other important points. The fact that it was conducted using only one type of stent, the Ankura, could lead to biased results. Additionally, the small cohort and physician references could lead to significant bias in the findings. Future studies should take these limitations into account and focus on a broader perspective, including a larger patient population and long-term outcomes.

## 5. Conclusions

It has been observed that advancements in endovascular techniques and technological designs can lead to successful outcomes in complex anatomical regions such as the aortic arch. Careful patient selection and detailed procedure planning restrict the use of the procedure in emergency cases. Surgeons should also remember that the relevant center must have significant experience in both open surgical and endovascular procedures. Despite all these challenges, our study is important in demonstrating that the in situ needle fenestration technique can be effective and feasible in cases of total aortic arch. However, it should be emphasized again that it needs to be supported by a larger patient population, multicenter studies, and long-term patient data.

## Figures and Tables

**Table 1:** Some patient demographics and clinical data.

Patients (n=15)	
**Sex**	
Male	12
Female	3
**Age**	
Mean	51.33 ± 19.69
Range	19–65
**Timing of treatment**	
Elective	15
Urgent	0
**Main pathology**	
Aortic aneurysm	5
Aortic dissection	10
**Clinical presentation**	
Symptomatic	4
Asymptomatic	11
**Comorbidities**	
Hypertension	6
Diabetes mellitus	2
Chronic kidney disease	1
Chronic pulmonary disease	1
Coronary artery disease	2
Peripheric vascular disease	3
**Smoking history**	6
**Prior aortic surgery**	10
**Stroke history**	0

**Table 2:** Perioperative Outcomes and Complications in the First 30 Days.

Mean operation time (minutes) (range)	197.33 (126−302)
0-30 days mortality (n,%)	1 (6.66%)
Total hospital duration (mean days, ± SD)	7 ± 3.36
Preoperative hemoglobin level (g/dL)	13.58 ± 2.11
Postoperative hemoglobin level (g/dL)	10.92 ± 2.08
Preoperative creatinine level (mg/dL)	0.98 ± 0.25
Postoperative creatinine level (mg/dL)	1.16 ± 0.32
Major adverse events in 30 days (n)	
Stroke	0
Transient ischemic attack	1
Spinal cord ischemia	0
Necessity of reintervention (n,%)	8 (53.3%)
Access related complications (n,%)	2 (13.3%)

n = number, SD = Standard Deviation.

## Data Availability

The original contributions presented in this study are included in this article; further inquiries can be directed to the corresponding authors.
